# The role of mediastinal adipose tissue 11β-hydroxysteroid d ehydrogenase type 1 and glucocorticoid expression in the development of coronary atherosclerosis in obese patients with ischemic heart disease

**DOI:** 10.1186/1475-2840-11-115

**Published:** 2012-09-25

**Authors:** Fatmahan Atalar, Selcuk Gormez, Baris Caynak, Gokce Akan, Gamze Tanriverdi, Sema Bilgic-Gazioglu, Demet Gunay, Cihan Duran, Belhhan Akpinar, Ugur Ozbek, Ahmet Sevim Buyukdevrim, Zeliha Yazici

**Affiliations:** 1 Department Growth-Development and Pediatric Endocrinology, Child Health Institute, Istanbul University, Istanbul, Turkey; 2 Department of Cardiology, Acibadem Kadikoy Hospital, Istanbul, Turkey; 3 Department of Cardiovascular Surgery, Istanbul Bilim University, Istanbul, Turkey; 4 Department of Medical Biology and Genetics, Istanbul Bilim University, Istanbul, Turkey; 5 Department of Histology and Embryology, Istanbul University, Cerrahpasa Medical School, Istanbul, Turkey; 6 Department of Immunology, Institute for Experimental Medical Research, Istanbul University, Istanbul, Turkey; 7 Biochemistry Laboratory, Florence Nightingale Hospital, Istanbul, Turkey; 8 Department of Radiology, Istanbul Bilim University, Istanbul, Turkey; 9 Department of Genetics, Institute for Experimental Medical Research, Istanbul University, Istanbul, Turkey; 10 Emeritus, Department of Internal Medicine, Section of Diabetes and Allied Disorders, Istanbul School of Medicine, Istanbul University, Istanbul, Turkey; 11 Department of Pharmacology, Istanbul Cerrahpasa Medical School, Istanbul University, Istanbul, Turkey

**Keywords:** Mediastinal adipose tissue, Glucocorticoid, Inflammation, Coronary artery disease, Stearidonic acid, Cortisol

## Abstract

**Background:**

Visceral fat deposition and its associated atherogenic complications are mediated by glucocorticoids. Cardiac visceral fat comprises mediastinal adipose tissue (MAT) and epicardial adipose tissue (EAT), and MAT is a potential biomarker of risk for obese patients.

**Aim:**

Our objective was to evaluate the role of EAT and MAT 11beta-hydroxysteroid dehydrogenase type 1 (11β-HSD-1) and glucocorticoid receptor (GCR) expression in comparison with subcutaneous adipose tissue (SAT) in the development of coronary atherosclerosis in obese patients with coronary artery disease (CAD), and to assess their correlations with CD68 and fatty acids from these tissues.

**Methods and results:**

Expression of 11β-HSD-1 and GCR was measured by qRT-PCR in EAT, MAT and SAT of thirty-one obese patients undergoing coronary artery bypass grafting due to CAD (obese CAD group) and sixteen obese patients without CAD undergoing heart valve surgery (controls). 11β-HSD-1 and GCR expression in MAT were found to be significantly increased in the obese CAD group compared with controls (p < 0.05). In the obese CAD group, 11β-HSD-1 and GCR mRNA levels were strongly correlated in MAT. Stearidonic acid was significantly increased in EAT and MAT of the obese CAD group and arachidonic acid was significantly expressed in MAT of the obese male CAD group (p < 0.05).

**Conclusions:**

We report for the first time the increased expression of 11β-HSD-1 and GCR in MAT compared with EAT and SAT, and also describe the interrelated effects of stearidonic acid, HOMA-IR, plasma cortisol and GCR mRNA levels, explaining 40.2% of the variance in 11β-HSD-1 mRNA levels in MAT of obese CAD patients. These findings support the hypothesis that MAT contributes locally to the development of coronary atherosclerosis via glucocorticoid action.

## Background

In obesity, fat depots localized around the heart are reported to contribute to the pathogenesis of coronary artery disease (CAD) [1-3]. Epicardial adipose tissue (EAT) generates various bioactive molecules which significantly affect cardiac function, and their association with the severity of CAD was has also been reported [2,4]. Human and animal studies have demonstrated EAT is located near to the arteries and that segments of an artery surrounded by EAT develop atherosclerosis faster than the intra-myocardial segments of the same artery [5]. Despite the fact that mediastinal adipose tissue (MAT, also known as paracardial adipose tissue or intrathoracic adipose tissue) is not close to the coronary arteries, it constitutes the majority of cardiac fat. Interestingly, a recent magnetic resonance imaging (MRI) study has shown that MAT contributes to the development of cardiovascular diseases [6,7]. Moreover, the presence of an association between cardiac visceral fat mass (epicardial and mediastinal) and insulin resistance, blood pressure and CAD has been demonstrated by MRI and computed tomography (CT) scans [3,8,9]. Visceral fat depots are recognized as a rich source of 11beta-hydroxysteriod dehydrogenase type 1(11β-HSD-1) and adipokines such as adiponectin, resistin, inflammatory cytokines, glucocorticoids and free fatty acids (FFA) [10,11]. Their important role in the pathogenesis of visceral obesity has also been demonstrated by clinical and animal studies [12-17]. The deposition and distribution of visceral fat, and associated atherogenic complications, are known to be mediated by glucocorticoids and 11β-HSD-1 activity, whilst an increased level of cortisol is also reported in obese subjects [18]. This increased cortisol, produced through 11β-HSD-1, in turn feeds back on visceral fat to stimulate 11β-HSD-1 and to cause additional visceral fat production. In addition, the expression level of glucocorticoid receptor in intra-abdominal fat and epicardial fat were shown to be upregulated in obesity and CAD leading to an amplification of glucocorticoid signaling and the expansion of these fat depots [19,20]. The question of whether the cardiac visceral adipose tissues (EAT and MAT) and SAT have similar 11β-HSD-1 and glucocorticoid receptor (GCR) gene expression patterns in obese patient with CAD (obese CAD group) and non-CAD (controls) has not been addressed before. Therefore, we aimed to compare the expression of the two major determinants of glucocorticoid action; 11β-HSD-1, known as the cortisol regenerating enzyme, and GCR in two different cardiac visceral fat depots in addition to the subcutaneous fat depots of the obese CAD group and controls. This was performed in order to evaluate their contribution to the development of the coronary atherosclerosis and to study their association with metabolic and clinical parameters and fatty acid profiles from these tissues.

## Methods

### Subjects

There were thirty-one obese patients undergoing coronary artery bypass grafting (CABG) due to CAD (obese CAD group) and sixteen obese patients without CAD undergoing heart valve surgery (controls). All patients were evaluated by a multidisciplinary team and met the appropriate criteria for the operation. All the patients with CAD had angiographic evidence of critical coronary atherosclerosis involving three vessels deemed necessary for an elective CABG surgery. Subjects with any history of HIV, viral hepatitis, cancer, collagen diseases, endocrinopathies, secondary hypertension and diabetic microangiopathic complications were excluded from the study. None of the female patients were on birth control pills or postmenopausal hormone replacement. Controls did not show any atherosclerotic lesions in their coronary angiographies. A detailed history was obtained, and physical examination was done for each subject. Blood pressure, height, weight, hip and waist circumference were recorded. BMI was calculated for all the patients as weight in kilograms divided by the square of height in meters. Obesity was identified according to BMI using the National Institutes of Health (NIH) criteria. The mean BMI of our study group, which comprised of obese CAD group and controls, was 30.2 kg/cm^2^. The cut-off points of waist circumference for cardiovascular disease risk were 87 cm for men and 83 cm for women, as defined by the criteria of TEKHARF (Turkish Adults Heart Disease and Risk Factors Study) [21]. Diagnosis of hypertension was based on a resting systolic or diastolic blood pressure >140 or >90 mmHg respectively, or current use of antihypertensive therapy. Dyslipidemia was defined as low-density lipoprotein (LDL) cholesterol ≥3 mmol/l or the use of hypolipidemic agents. All patients had normal dietary regimes at least three days before taking blood samples. Plasma and tissue samples were obtained after 10 hours (overnight) fasting. Fasting venous blood samples were separated as serum, plasma and cellular portions and stored at −80°C for biochemical analyses. EAT, MAT and SAT were collected during CABG and heart valve surgeries in the form of biopsy. Tissue samples were then immediately frozen in liquid nitrogen and stored at −80°C prior to total RNA preparation and fatty acid analysis. Oral antidiabetics, including metformin, and lipid lowering drugs which may interfere with adipocytokine gene expression were stopped 3 days before blood and tissue sample collection. None of the patients were treated with thiazolidinediones. All patients gave their written informed consent. The project was approved by the local ethics committee of Istanbul Science University and was performed in accordance with the ethical standards formulated in the Helsinki Declaration.

### Biochemical measurements

Serum total cholesterol and high-density lipoprotein (HDL) cholesterol, low-density lipoprotein (LDL) cholesterol, triglycerides (TG), fasting glucose, fasting insulin and morning plasma cortisol were measured by routine assays in the hospital laboratory. All analyses were performed on Cobas 6000 instrument (Roche). Insulin resistance was evaluated using the homeostatic model assessment of insulin resistance (HOMA-IR = fasting glucose [mmol/L]x fasting insulin[uU/mL]/22.5). A HOMA-IR value greater than 2.24 as suggested by the TEKHARF study was taken as the cut-off point for insulin resistance for the study group [22]. Body fat amount was assessed by the bioelectrical impedance technique (MC 180, TANITA Corporation, Tokyo, Japan). Volumetric measurement of epicardial and subcutaneous fat were obtained by multislice computed tomography (LightSpeed VCT 64, GE Healthcare or Aquillon 16, Toshiba Medical Systems, Tokyo, Japan).

### Tissue biopsies

Paired sample biopsies of adipose tissues from 3 compartments, namely epicardial, mediastinal, and subcutaneous, were taken from the chest of each individual during surgery. Epicardial adipose tissue (EAT) corresponds to the adipose depot in direct contact with the heart located between the myocardium and the visceral pericardium. Mediastinal adipose tissue (MAT) was defined as the fat deposited in intrathoracic space and subcutaneous adipose tissue (SAT) as the fat volume located anterior to the sternum and posterior to the vertebra. Immediately after resection, approximately 100–300 mg of each fat tissue was snap-frozen in liquid nitrogen. All tissue samples were then stored at −80°C until RNA isolation and fatty acid analysis.

### Total RNA extraction and quantitation

The EAT, MAT and SAT were disrupted with a homogeniser in the reaction tube. Total RNA from EAT, MAT and SAT were extracted with the Allprep kit (Qiagen, Germany). RNA quantity was determined by Picodrop (Picodrop, Saffron Walden, UK). After DNase I treatment, 1 μg of RNA was reverse transcribed in 20 μl total volume using random hexamers and poly(dt) as primers and Superscript II reverse transcriptase (Invitrogen). Following cDNA synthesis, the quality of cDNA was assessed by PCR amplification of the housekeeping gene beta-globin. cDNA was stored at −80°C until required.

### Gene expression analysis of 11Î²-HSD-1, GCR and CD68

mRNA expression levels of 11β-HSD-1, GCR and CD68 were determined by SYBR green-based qRT-PCR using a LightCycler (Roche-Germany) instrument. Ten fold dilutions of cDNA synthesized from total RNA were used. All samples were amplified in duplicate and the mean was obtained for further calculations. Primers were designed using the software Primer3 v.0.4.0 (http://frodo.wi.mit.edu/primer3/) and synthesized by TIB-MOLBIOL (Berlin-Germany).

### Immunohistochemical staining

Epicardial, mediastinal and subcutaneous tissue samples from obese CAD group and control group were studied in order to demonstrate the presence of CD68 expression. Epicardial, mediastinal and subcutaneous tissue samples taken during the operation were immediately fixed in 10% neutral buffered formalin, then following the dehydration they were embedded in paraffin. Tissue-sections (5 μm thick) were taken with microtome (Leica SM 2000, Germany) on poly-L-lysine-coated slides and dried at room temperature. The tissue-sections were then deparaffinized in xylene and rehydrated in graded alcohol. Zymed Histostain-plus-Peroxidase-kit (Zymed Laboratoratories Inc.,USA, prepared according to manufacturer’s instructions) protocols were applied. Sections were then washed with distilled water and phosphate buffered saline (PBS) for 10 minutes and then treated with 3% H_2_O_2_ for 10 minutes to inhibit endogenous peroxidase activity. Then, sections were incubated with primary antibody directed against CD 68 (CD 68, 1:100 dilution; Santa Cruz, Lot#J2207) for 18 hours at 4*°*C in a humidity chamber. On the following day, sections were incubated with biotinylated secondary antibody and then with streptavidin conjugated to horseradish peroxidase (both from Zymed Histostain-plus-Peroxidase-kit, Zymed Laboratoratories Inc.,USA, prepared according to manufacturer’s instructions). Finally, to reveal the immunolabelling, sections were incubated with AEC Substrate Kit (Zymed Laboratoratories Inc., USA) for 5 minutes and then counterstained with Mayer’s hematoxylin and the samples were photoghraphed by Olympus DP72 model digital microscope cameras.

### Determination of free fatty acids in serum and tissues

Fatty acid profiles were prepared by a slight modification of a method previously described by Yazici et al. [23]. Each sample was weighed and then homogenized in cold 154 mM NaCl. Total lipids and added internal standard (100 μg nonadecanoic acid in chloroform, Sigma Chemical Co., St.Louis) were extracted with chloroform/methanol (2:1) containing 0.005% butylated hydroxytoluene. The chloroform phase was removed and evaporated to dryness under a stream of nitrogen. Total lipids were saponified with 2% KOH in methanol and the fatty acids methylated with hexane and analyzed by capillary gas chromatography (Perkin-Elmer 8420 Capillary Gas Chromatography, Gouda, The Netherlands, Column: 50x 0.25 mm WCOT fused silica, CP-sil 88; flame-ionization detector temperature 300°C; oven temperature from 150 to 230°C at 2°C min^-1^; carrier gas N_2_). The mass spectra of FAME from representative samples were obtained using a Hewlett Packard (HP) 6890 capillary GC interfaced with a HP mass selective detector and controlled by a HP Chem station (Column: 25x 0.25 mm ID, QC2 x BPx70; detector temperature 280°C; oven temperature programme from 100°C to 290°C at 3°C min^-1^; carrier gas helium).

FAMEs were identified by their retention time and by comparison with those of authentic standards (Sigma Chemical Co., St. Louis), and by GC-Mass Spectrometry. The detector response factors were determined by injecting equal amounts of fatty acids and internal standard methyl esters on the column. Their amounts were estimated by calculating the corresponding areas of fatty acid and internal standard.

### Data analysis

Gene expression data were obtained as Ct values (Ct = the cycle number at which logarithmic PCR plots cross a calculated threshold line). Ct values were used to calculate ΔCT values (ΔCT = Ct of the target gene minus Ct of the housekeeping gene). The expression of each gene was compared between depots using the ΔΔCt method. All statistical analysis was performed at the ΔCt stage in order to exclude potential bias due to averaging of data transformed through the equation 2^-ΔΔCt^. Target gene expression was calculated by using the expression of a housekeeping gene (cyclophilin) as an internal standard. The presence of specific gene products was also confirmed with melting curve analysis. Data are expressed as numbers and percentages for discrete variables and as mean ± SD, for continuous variables. Baseline differences between obese CAD group and controls were examined by Kruskall Wallis and Mann–Whitney *U* test. Variables were compared by Spearman’s correlation to be able to eliminate the effect of outliers. Multiple linear regression was used to estimate the risk of increased MAT 11β-HSD-1, GCR and CD68 expression for developing atherosclerosis in obese patients with CAD. For the analysis, increased expression levels of genes in MAT were used as dependent variables and plasma cortisol, HOMA-IR and stearidonic acid as independent variables. These results are reported as coefficient of determination (R^2^), which indicates the percentage of variation in the dependent variable that can be explained by the independent variables. Statistical significance was taken as p < 0.05.

## Results

### Patient data

The anthropometric, clinical and metabolic characteristics of obese CAD and control groups are shown in Table 1. LDL cholesterol (*p < 0.05*), cortisol (*p < 0.05*), HOMA-IR (*p < 0.05*) and epicardial fat volume (*p < 0.001*) were significantly different in obese CAD group compared with controls. CAD risk factors including smoking and family history of CAD were also significantly different between groups (*p < 0.05* and *p < 0.001*, respectively).

**Table 1:** The antropometric, clinical and metabolic data of the study group

	**Obese CAD group (n = 31)**	**Controls (n = 16)**	**p value**
***Antropometric characteristics***			
**Age (years)**	57.4 ± 9.1	58.1 ±10.4	NS
**Female–male (%)**	66-34	51-49	NS
**BMI (kg/m**^**2**^**)**	33.6 ± 3.1	30.0 ±5.2	NS
**Waist/hip ratio**	1.1 ± 0.0	0.8 ± 0.1	NS
***Risk factors***			
**Smoking (%)**	44	66	***0.004***
**Family history of CAD (%)**	62	25	***0.0001***
***Metabolic characteristics and indices***			
**SBP (mm Hg)**	135.9 ± 19.4	129.5 ± 30.2	NS
**DBP (mm Hg)**	78.5 ± 8.9	77.3 ± 8.1	NS
**Total-cholesterol (mg/dl)**	196.1 ± 31.9	200.1 ± 31.4	NS
**HDL-cholesterol (mg/dl)**	41.2 ± 9.0	45 ± 11.9	NS
**LDL-cholesterol (mg/dl)**	111.5 ± 19.0	142.4 ± 29.7	***0.041***
**Triglycerides (mg/dl)**	191.0 ± 21.5	201.1 ± 24.1	NS
**Cortisol (μg/dl)**	13.1 ± 4.5	8.2 ± 3.1	***0.006***
**HOMA-IR**	3.1 ± 1.6	1.9 ± 1.0	***0.005***
**C-peptid (ng/ml)**	2.6 ± 1.1	3.2 ± 1.2	NS
**Epicardial fat (cm**^**3**^**)**	8.8 ± 5.1	6.0 ± 2.5	***0.001***
**Subcutaneous fat (cm**^**3**^**)**	138.8 ± 44.1	133.1 ± 39.1	NS

Values are represented as mean ± SD, or as percentages. SD: Standard deviation, BMI: Body mass index, SBP: Systolic blood pressure, DBP: Diastolic blood pressure, HDL: High density lipoprotein, LDL: Low density lipoprotein, HOMA-IR: Homeostasis Model of Assessment - Insulin Resistance. Groups were compared with Mann–Whitney *U* test for the variables.

### 11Î²-HSD-1 and GCR expressions in EAT, MAT and SAT

**Figure 1:**
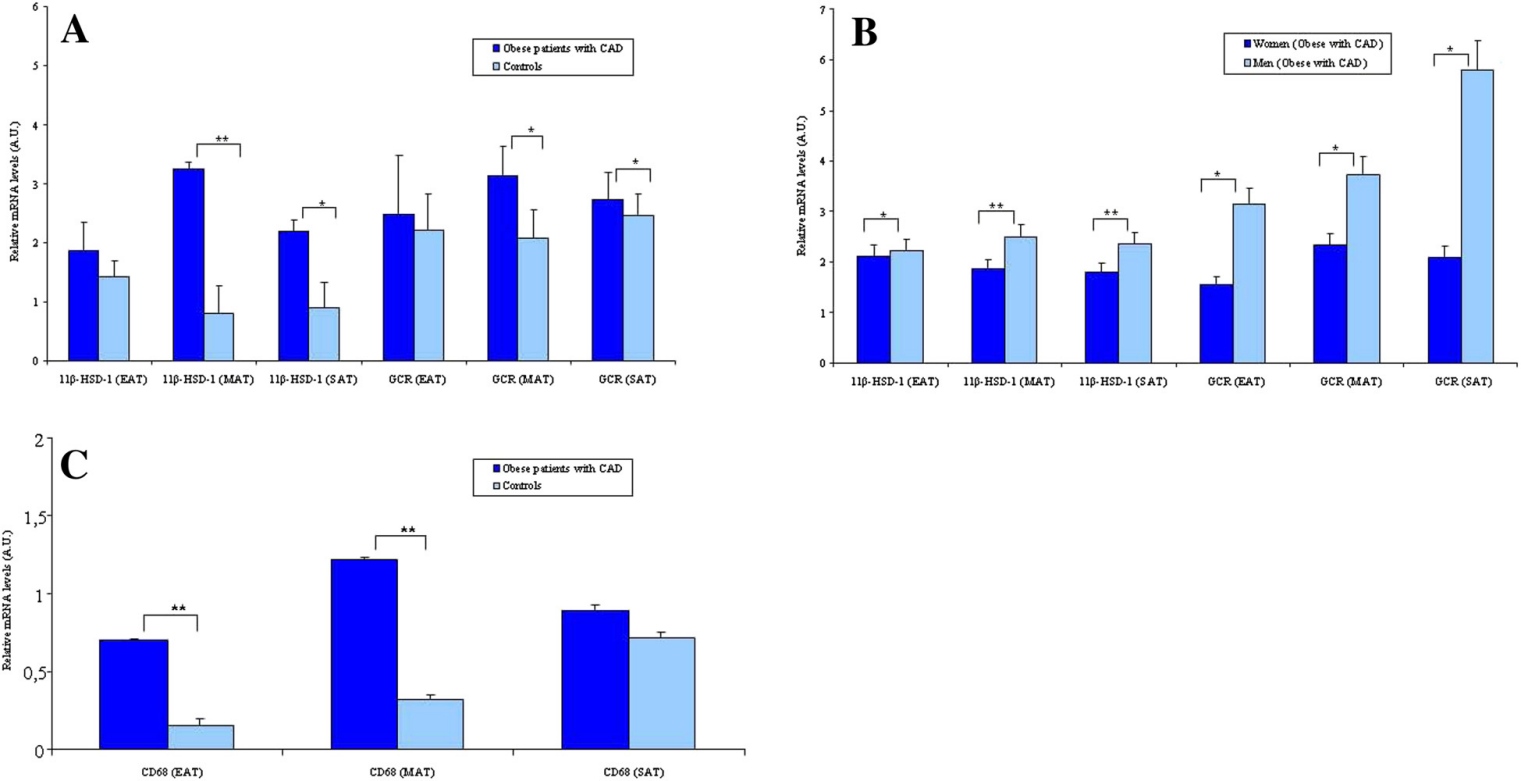
**The mRNA expression of 11β-HSD1 and GCR in the study groups. A**: The mRNA expression of 11β-HSD1 and GCR in 31 obese patients with CAD (obese CAD group) and 16 obese patients without CAD (control group) in epicardial adipose tissue (EAT), mediastinal adipose tissue (MAT) and subcutaneous adipose tissue (SAT). *p < 0.05, **p < 0.001 (Obese CAD group vs.control group). Data are mean ± SD. **B**: The mRNA expression of 11β-HSD1 and GCR in 16 females and 15 males obese patients with CAD in epicardial adipose tissue (EAT), mediastinal adipose tissue (MAT) and subcutaneous adipose tissue (SAT). * p < 0.05, **p < 0.001 (women vs.men). Data are mean ± SD, **C**: The mRNA expression of CD68 in 31 obese patients with CAD (obese CAD group) and 16 obese patients without CAD (control group) in epicardial adipose tissue (EAT), mediastinal adipose tissue (MAT) and subcutaneous adipose tissue (SAT). **p < 0.01 (Obese CAD group vs.control group). Abbreviations: A.U., Arbitrary Units.

EAT, MAT and SAT depots were assessed for expression of 11β-HSD-1 and GCR by qRT-PCR in the study group (Figure 1A). We found that 11β-HSD-1 and GCR mRNA levels of obese CAD group were significantly higher in MAT compared to EAT and SAT (*p < 0.05*, respectively), and, 11β-HSD-1 mRNA levels in MAT and SAT were significantly different in obese CAD group compared to controls (*p < 0.05 and p < 0.001,* respectively). In addition, GCR mRNA levels in MAT and SAT were found to be significantly higher in obese CAD group compared to controls (*p < 0.05,* respectively). Furthermore, the expression levels of 11β-HSD-1 and GCR were further evaluated in both sexes. Men had significantly higher expression of 11β-HSD-1 and GCR in EAT, MAT and SAT when compared to women (Figure 1B). 11β-HSD-1 and GCR gene expression in EAT, MAT and SAT of females from the controls was no different than males (data not shown).

### CD68 mRNA expressions in EAT, MAT and SAT

**Figure 2:**
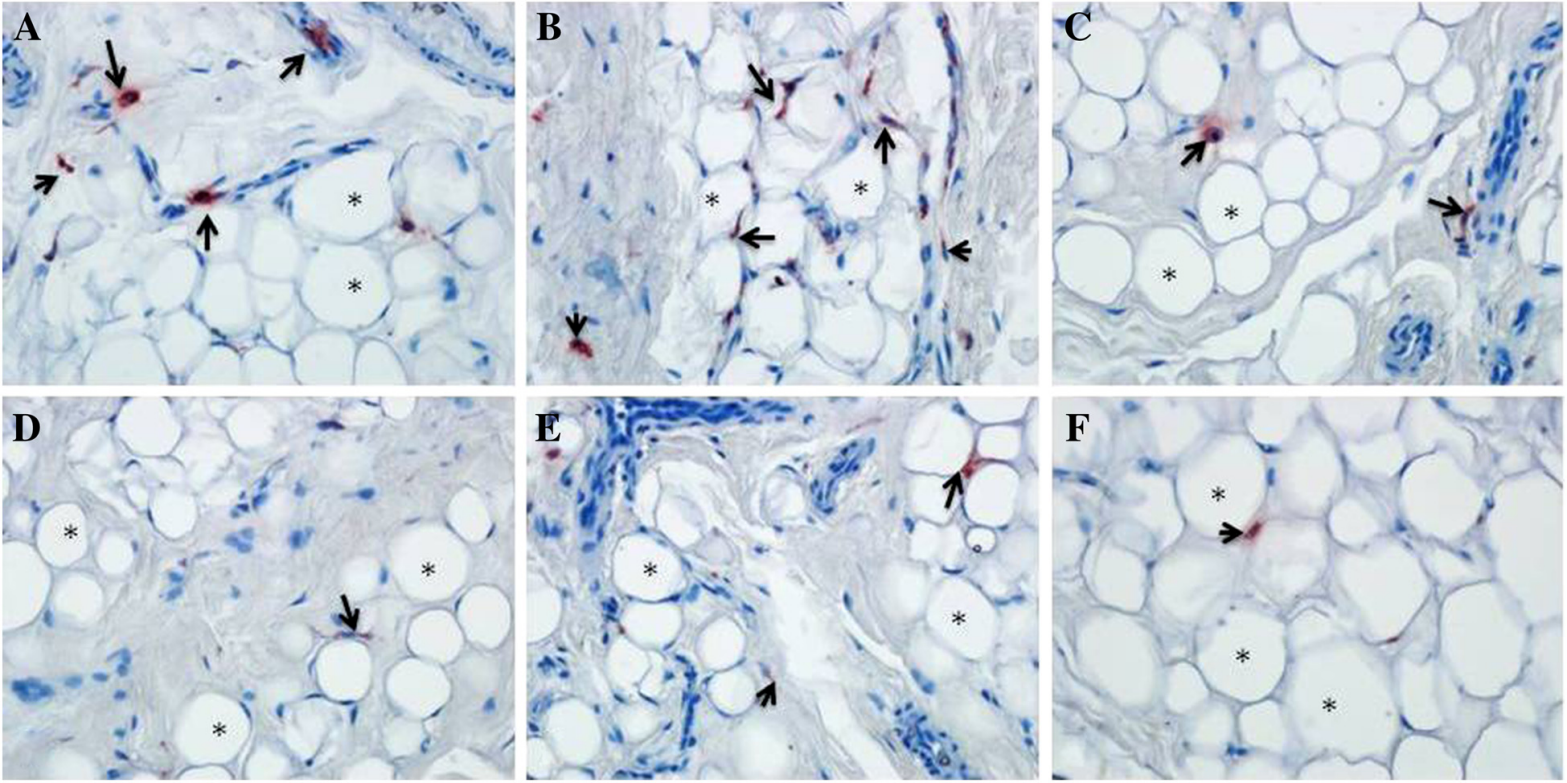
**Immunohistochemistry using human anti-CD68 for macrophages for epicardial, mediastinal and subcutaneous adipose tissues of obese CAD and control groups.** CD68+ cells (macrophages) are observed in the epicardial, mediastinal and subcutaneous adipose tissues of obese CAD group (**A**, **B** and **C**) and control group (**D**, **E** and **F**). CD68+ cells (upwards arrow) between normal and large adipocytes (black asterisks) are identified in the adipose tissues. These findings were endorsed by qRT-PCR of CD68 mRNA expression in all three adipose tissues of the study group. Magnification: x 40 for **A**, **B**, **C**, **D**, **E** and **F.**

The significant effect of infiltrating macrophages on adipokine expressions from adipose tissue is well known, therefore we evaluated mRNA expression of CD68; a macrophage marker, in EAT, MAT and SAT in both groups. As shown in Figure 1C, the mRNA levels of CD68 in MAT and EAT were found to be significantly higher in obese CAD group compared to controls (*p < 0.05,* respectively). MAT CD68 mRNA levels of obese CAD group were almost two fold higher compared to EAT and SAT. In parallel with mRNA expression analysis results, the immunohistochemical analysis also demonstrated the presence of increased CD68+ cells in MAT of CAD group. Representative photomicrographs showing CD68+ cells stainings in three adipose tissues of obese CAD group and control group are shown in Figure 2.

The obese CAD patients with a waist circumference greater than the cut-off values (cut-off points of waist circumference for cardiovascular disease risk were 87 cm for men and 83 cm for women) had increased levels of C:18:4n-3, CD68, 11β-HSD-1 and GCR in MAT (p < 0.05, respectively). In addition, obese CAD patients with HOMA-IR above the cut-off value of 2.24 showed a strong association with increased CD68 and GCR mRNA levels in EAT and MAT (p < 0.05, respectively).

### Correlations between gene expression levels and antropometric parameters and fat volumes

**Figure 3:**
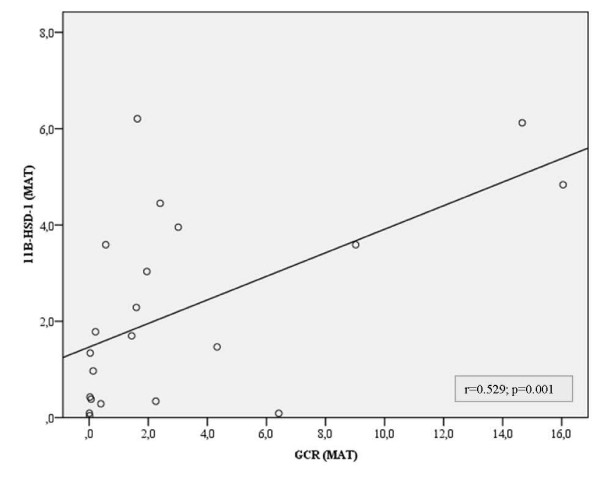
**Scatter plot of 11β-HSD1 versus GCR in mediastinal adipose tissue (MAT) of obese CAD group.** Correlation between mRNA levels of 11β-HSD1 and GCR were assessed using Spearman’s correlation.

Table 2 shows the correlation of 11β-HSD-1 and GCR mRNA levels in EAT, MAT and SAT depots with study variables for obese CAD group. A positive correlation was determined between EAT and MAT GCR mRNA levels and waist circumference (r = 0.520; *p = 0.002*, r = 0.4; *p = 0.019*) and between SAT GCR mRNA levels and BMI (r = 0.429; *p = 0.009*). We demonstrated that 11β-HSD-1 mRNA levels in EAT, MAT and SAT were positively correlated with weight (r = 0.442; *p = 0.006*, r = 0.340; *p = 0.027*, r = 0,570; *p = 0.0001*). 11β-HSD-1 mRNA levels in EAT was found to be correlated with epicardial fat volume (r = 0.447; p = *0.009*). In obese CAD group, EAT 11β–HSD-1 mRNA expression were correlated with the expression in MAT and SAT (r = 0.398; *p = 0.015*, r = 0.574; *p = 0.0001*, respectively). Importantly, a positive correlation was determined between 11β-HSD-1 and GCR mRNA levels in MAT (r = 0.529; *p = 0.001*) (Figure 3).

**Table 2:** Spearman correlation matrix of gene expression of 11β-HSD1 and GCR in epicardial (EAT), mediastinal (MAT) and subcutaneous (SAT) adipose tissues with study variables for obese CAD group (n = 31)

**Variables**	**11 β-HSD1 (EAT)**		**11 β-HSD1 (MAT)**		**11 β-HSD1 (SAT)**		**GCR (EAT)**		**GCR (MAT)**		**GCR (SAT)**	
	**r**	**p**	**r**	**p**	**r**	**p**	**r**	**p**	**r**	**p**	**r**	**p**
Weight	0.442	0.006	0.340	0.027	0.570	0.000	0.133	0.666	0.691	0.009	0.492	0.088
BMI	0.159	0.363	0.269	0.118	0.152	0.384	0.314	0.07	0.317	0.060	0.429	0.009
Waist circumference	0.017	0.926	0.244	0.171	0.1	0.58	0.520	0.002	0.400	0.019	0.239	0.189
Fat (kg)	0.249	0.15	0.248	0.151	0.233	0.177	0.261	0.135	0.236	0.18	0.285	0.102
Epicardial fat (cm^3^)	0.447	0.009	0.240	0.179	0.330	0.061	0.121	0.501	0.146	0.418	0.202	0.256
Subcutaneous fat (cm^3^)	0.068	0.702	0.207	0.239	0.117	0.510	0.207	0.240	0.243	0.167	0.176	0.318
11 β-HSD1 (EAT)			0.398	0.015	0.574	0.000	0.394	0.018	0.292	0.084	0.381	0.022
11 β-HSD1 (MAT)					0.393	0.016	0.471	0.004	0.529	0.001	0.400	0.16
11 β-HSD1 (SAT)							0.358	0.032	0.374	0.025	0.335	0.046
GCR (EAT)									0.496	0.002	0.389	0.01
GCR (MAT)											0.580	0.000

### Fatty acid analysis of serum and adipose tissues

In MAT and EAT, C:18:4n-3 (also known as *all-cis*-6,9,12,15,-octadecatetraenoic acid or stearidonic acid) and n-6/n-3 were found to be significantly higher in obese CAD group compared controls *(p < 0.05)* (Table 3). In EAT only, C:16:1n-7 (palmitoleic acid), C:20:3n-6 (eicosatrienoic acid) and n-3 ratio were also significantly different between the groups (*p < 0.05,* respectively). In MAT, stearidonic acid was correlated with 11β-HSD-1 and GCR mRNA levels (r:0.536; *p = 0.038*, r = 0.682; *p = 0.007*, respectively). Furthermore in MAT, 20:4n-6 (arachidonic acid) levels were also significantly higher in male compared to female obese patients with CAD and n-6/n-3 ratio was found to be significantly lower in obese CAD group compared to controls *(p < 0.05*, respectively).

**Table 3:** Fatty acid composition of serum, EAT, MAT and SAT of obese CAD group (n = 15) and controls (n = 5)

	**In SERUM**				**In EAT**			**In MAT**			**In SAT**	
**Fatty acids**	**Obese CAD group (n = 15)**	**Controls (n = 5)**	**P value**	**Obese CAD group (n = 15)**	**Controls (n = 5)**	**P value**	**Obese CAD group (n = 15)**	**Controls (n = 5)**	**P value**	**Obese CAD group (n = 15)**	**Controls (n = 5)**	**P value**
14:0 (myristic acid)	32.7 ± 4.8	32.3 ± 7.9	NS	9.6 ± 0.8	11.9 ± 1.1	NS	10 ± 0.9	9.7 ± 1.4	NS	10.1 ± 0.7	10.4 ± 0.7	NS
16:0 (palmitic acid)	625.2 ± 99.7	419.6 ± 121.3	NS	108.4 ± 7.1	113.9 ± 10	NS	103.7 ± 5.7	98.3 ± 11.8	NS	105.9 ± 7.1	115.6 ± 6.8	NS
16:1n-7 (palmitoleic acid)	67.1 ± 12.6	51.6 ± 12.7	NS	8.3 ± 0.8	14.6 ± 3.3	***0.017***	8.9 ± 0.8	6.7 ± 2	NS	10.7 ± 1.2	9.8 ± 2.9	NS
18:0 (stearic acid)	182.8 ± 18.3	169.2 ± 22.8	NS	26.5 ± 1.9	24.6 ± 4.5	NS	23.4 ± 1.6	23.9 ± 4.8	NS	22.5 ± 1.9	24 ± 3.6	NS
18:1n-9 (oleic acid)	671.8 ± 115.3	478.4 ± 71.4	NS	168.4 ± 17.5	172.7 ± 13.5	NS	178 ± 14.5	175.9 ± 18.2	NS	201.2 ± 15	217.6 ± 10.4	NS
18:2n-6 (linoleic acid)	834.7 ± 100.9	727.4 ± 104.3	NS	103.2 ± 6.8	105.8 ± 17.6	NS	106.4 ± 5.5	99.1 ± 17.5	NS	118 ± 8.8	117 ± 11.7	NS
18:3n-3 (linolenic acid)	30.2 ± 6.4	23.3 ± 6.6	NS	1.1 ± 0.3	0.4 ± 0.4	NS	0.6 ± 0.2	0.1 ± 0.1	NS	0.6 ± 0.2	0.2 ± 0.2	NS
18:4n-3 (octatetradecanonic acid)	N.D	N.D	NS	2.8 ± 0.3	1.4 ± 0.6	***0.026***	2.2 ± 0.4	0.3 ± 0.3	***0.026***	2.1 ± 0.4	2.1 ± 0.8	NS
20:1n-9 (eicosenoic acid)	N.D	N.D	NS	5.9 ± 0.6	5 ± 0.2	NS	6.6 ± 0.6	5.1 ± 0.8	NS	6 ± 0.5	6 ± 0.6	NS
20:3n-6 (eicosatrienoic acid)	40.3 ± 5.6	42.5 ± 8.3	NS	2.0 ± 0.1	1.3 ± 0.4	***0.038***	1.6 ± 0.2	1.4 ± 0.3	NS	2.2 ± 0.2	2.3 ± 0.6	NS
20:4n-6 (arachidonic acid)	136.8 ± 18	131 ± 19.4	NS	2.5 ± 0.3	1.9 ± 0.5	NS	1.7 ± 0.2	1.6 ± 0.3	NS	2.5 ± 0.3	1.8 ± 0.5	NS
20:5n-3 (eicosapentanoic acid)	N.D	N.D	NS	N.D	N.D	NS	N.D	N.D	NS	0.4 ± 0.3	0.2 ± 0,1	NS
22:5n-3 (dokosapentaenoic acid)	N.D	N.D	NS	1.4 ± 0.3	0.9 ± 0.4	NS	1.2 ± 0.3	0.4 ± 0.2	NS	0.6 ± 0.2	0.4 ± 0.2	NS
22:6n-3 (docosahexaenoic acid)	N.D	N.D	NS	2 ± 0.4	1.3 ± 0.7	NS	2.8 ± 1.7	0.7 ± 0.4	NS	4.4 ± 1.8	1.8 ± 0.6	NS
24:1n-9 (nervonic acid)	25 ± 4.6	33.6 ± 11.4	NS	1.9 ± 0.4	0.6 ± 0.4	NS	1.6 ± 0.4	0.7 ± 0.3	NS	1.6 ± 0.3	1.2 ± 0.4	NS
n-3 (ω − 3)	35 ± 9.5	40.3 ± 9.2	NS	7.5 ± 0.6	4 ± 1.3	***0.016***	7 ± 1.7	1.6 ± 0.8	NS	8.1 ± 1.9	4.8 ± 0.6	NS
n-6 (ω − 6)	1011.7 ± 115.3	900.9 ± 125.4	NS	107.6 ± 7	109 ± 18.2	NS	109.6 ± 5.7	102 ± 17.7	NS	122.8 ± 9	121.2 ± 12	NS
n-9 (ω − 9)	681.8 ± 116.2	491.8 ± 76.4	NS	176.2 ± 18	178.4 ± 13.9	NS	186.2 ± 15	181.6 ± 19	NS	209 ± 15.2	224.9 ± 10.7	NS
Saturated	840.7 ± 119.9	726.4 ± 107	NS	144.6 ± 9.2	150.3 ± 15.3	NS	137.2 ± 16.9	132 ± 17.7	NS	138.5 ± 9.1	150.1 ± 10.6	NS
Unsaturated	1790.9 ± 237.4	1476.6 ± 205	NS	299.8 ± 20	306 ± 15.3	NS	311.7 ± 16.9	292 ± 36.8	NS	350.4 ± 19	360.5 ± 21	NS
Ratios												
16:0 / 16:1	11 ± 1.1	11.1 ± 1.1	NS	14.8 ± 2	10.1 ± 2.7	NS	12.6 ± 0.9	11.8 ± 1.8	NS	11 ± 1	12.6 ± 1.5	NS
18:0 / 18:1	0.3 ± 0.03	0.4 ± 0.05	NS	0.2 ± 0.02	0.14 ± 0.03	NS	0.14 ± 0.02	0.12 ± 0.02	NS	0.11 ± 0.01	0.11 ± 0.01	NS
18:2 / 20:4	6.7 ± 0.7	5.7 ± 0.6	NS	41.3 ± 3.6	47.1 ± 5.6	NS	64.4 ± 6.6	64.5 ± 13.5	NS	49.2 ± 5.3	49.4 ± 4.7	NS
n-6 / n-3	50.3 ± 9.6	24.1 ± 4.4	NS	16.0 ± 1.5	26.8 ± 6.3	***0.023***	19.5 ± 2.3	43.5 ± 15.4	***0.010***	19.1 ± 2.3	25.6 ± 4.2	NS
Saturated / Unsaturated	0.5 ± 0.02	0.5 ± 0.02	NS	0.5 ± 0.03	0.5 ± 0.04	NS	0.4 ± 0.02	0.4 ± 0.04	NS	0.4 ± 0.02	0.42 ± 0.03	NS

All values are mean percent of total ± SE. Groups were compared with Mann–Whitney *U* test for the variables. Significant value, p < 0.05 (Obese CAD group vs. controls). Abbreviations: EAT: epicardial adipose tissue MAT: mediastinal adipose tissue, SAT: subcutaneous adipose tissue.

### Linear multiple regression analysis

Analyses were performed to evaluate the relative contribution of biological parameters, related gene expressions and fatty acids to the relationship with MAT 11β-HSD-1, GCR and CD68 mRNA levels. The variables explaining the variance of MAT 11β-HSD-1, GCR and CD68 mRNA levels were evaluated in obese CAD group (Table 4). The analysis showed that the variables plasma cortisol, HOMA-IR, weight, stearidonic acid and MAT GCR mRNA levels could explain 40.2% of the variance in MAT 11β-HSD-1 mRNA levels in obese CAD group. In addition, MAT 11β-HSD-1 mRNA levels, weight and cortisol explained 35.1% of the variance in MAT GCR mRNA levels, MAT 11β-HSD-1, weight and GCR mRNA levels and cortisol explained 56.4% variance in MAT CD68 mRNA levels.

**Table 4:** Predictors of mediastinal adipokines mRNA expression in multiple linear regression analysis

**Mediastinal** **adipokines mRNA expressions**	**Variables**	**R**^**2**^	**Standardized β**	**P Value**
**11β–HSD-1 mRNA expression**	MAT GCR mRNA levels	0.402	0.448	*0.01*
	Cortisol		0.233	*0.029*
	C:18:4n-3		0.189	*0.064*
	HOMA-IR		−0.190	0.509
**GCR mRNA expression**	Weight	0.351	0.131	*0.039*
	MAT 11β–HSD-1 mRNA levels		0.785	*0.005*
	Cortisol		−0.501	*0.018*
	Weight		0.756	*0.038*
**CD68 mRNA expression**	MAT 11β–HSD-1mRNA levels	0.564	0.480	*0.004*
	MAT GCR mRNA levels		0.351	*0.019*
	Cortisol		0.250	*0.031*
	Weight		0.253	0.065

Multiple linear regression analysis with 11β–HSD-1, GCR and CD68 mRNA expression in mediastinal adipose tissue, cortisol, weight.

HOMA-IR and C:18:4n-3 as dependent variables and mediastinal adipokines mRNA expression in MAT as independent variables.

MAT: mediastinal adipose tissue; GCR: glucocortcoid receptor; C:18:4n-3: stearidonic acid. P < 0.05; statistical significance.

The variables explaining the variance of the mRNA levels of MAT 11β-HSD-1 were evaluated separately in women and men of the obese CAD group. In men, linear regression analysis with MAT 11β-HSD-1 mRNA level as the independent variable and plasma cortisol, weight, HOMA-IR, stearidonic acid and MAT GCR mRNA levels as the dependent variables showed only MAT GCR mRNA levels and stearidonic acid significantly explained the variation of 11β-HSD-1 (β = 0.746, p = *0.003*). In women, none of the variables explained the variance of the mRNA levels of 11β-HSD-1.

When the effects of age and sex were controlled with hierarchical multiple regression, MAT GCR mRNA levels and cortisol remained significant predictors (β = 0.768, p = *0.009*; β = 0.897, p = *0.032*, respectively) for MAT 11β-HSD-1 mRNA levels and they explained 31.2% of the variance in MAT 11β-HSD-1 mRNA levels. MAT 11β-HSD-1 mRNA levels remained the significant predictor (β = 0.926, p = *0.009*) for MAT GCR mRNA levels and explained the variance of 28% in MAT GCR expression. 11β-HSD-1 mRNA expression, GCR mRNA expression and cortisol remained significant predictors (β = 0.464, p = 0.005; β = 0.353, p = 0.019; β = 0.450, p = 0.057, respectively) for MAT CD68 mRNA levels and they explained the variance of 54.2% of CD68 expression of obese CAD group.

## Discussion

This study examined the relationships between the development of atherosclerosis and the two key determinants of tissue glucocorticoid action; 11β-HSD-1 and GCR, in cardiac fat depots of obese CAD group and controls. The major findings were; 1) 11β-HSD-1 and GCR mRNA levels of obese CAD group were significantly higher in MAT compared to EAT and SAT. In addition, 11β-HSD-1 and GCR mRNA levels in MAT and SAT were found to be significantly higher in obese CAD group compared to controls 2) CD68 mRNA expression was present in MAT and its expression was significantly higher in MAT compared to EAT and SAT. 3) MAT 11β-HSD-1 was positively correlated with MAT GCR mRNA levels in obese CAD patients 4) In MAT and EAT, stearidonic acid was significantly higher in obese CAD group compared to controls. And, in addition stearidonic acid in MAT was correlated with 11β-HSD-1 and GCR mRNA levels 5) Arachidonic acid was significantly higher in obese male CAD patients 6) Plasma cortisol was significantly increased in obese CAD group compared to controls. The regression analysis revealed that the interrelation between plasma cortisol, weight, stearidonic acid, MAT GCR mRNA levels along with HOMA-IR could explain 40.2% of the variance in MAT 11β-HSD-1 mRNA levels (dependent variable) in obese CAD group.

### Expression of adipokines and inflammatory cytokines are altered in MAT

**Figure 4:**
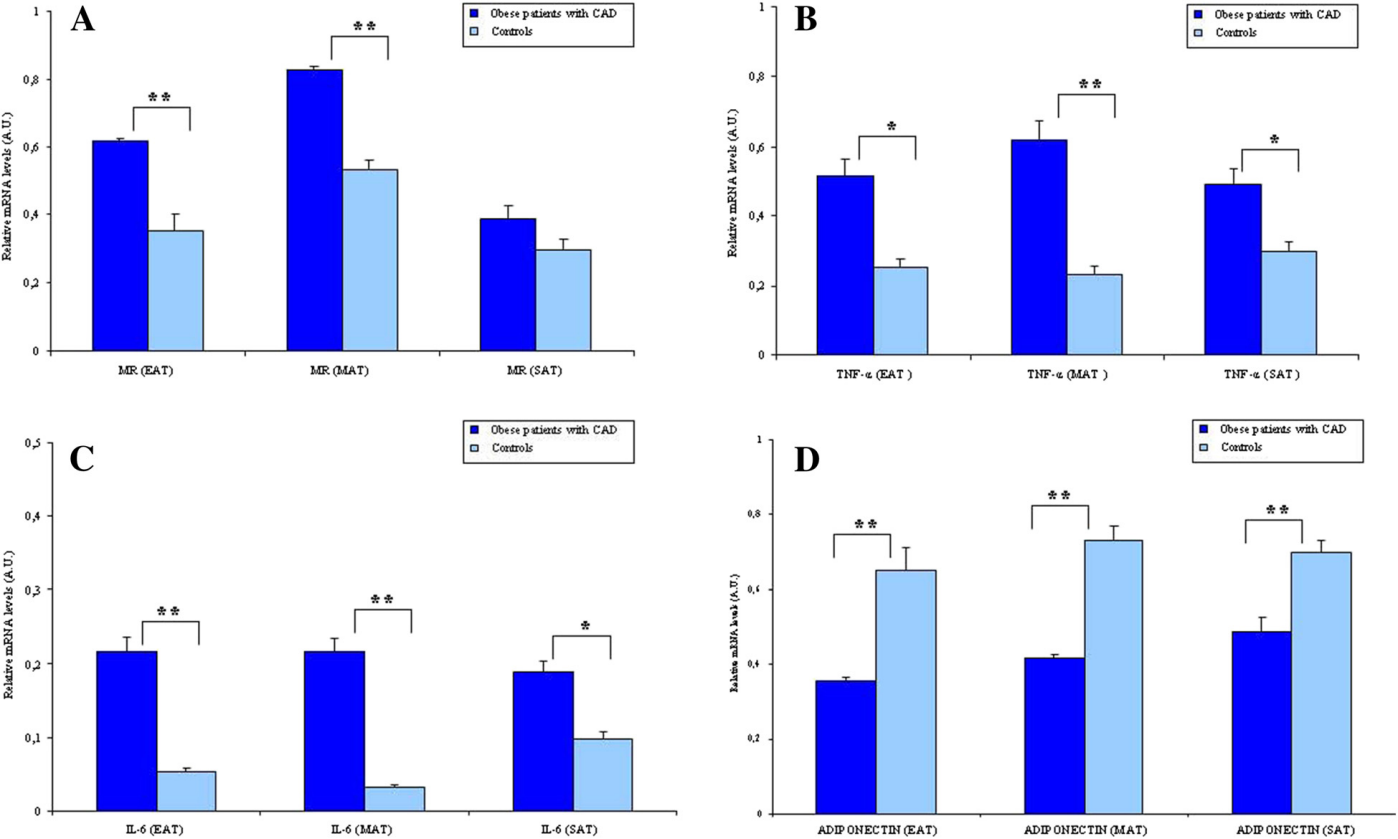
**The mRNA expression of Mineralocorticoid Receptor (MR) and TNF-α, IL-6 and adiponectin in 31 obese patients with CAD (obese CAD group) and 16 obese patients without CAD (control group) in epicardial adipose tissue (EAT), mediastinal adipose tissue (MAT) and subcutaneous adipose tissue (SAT).** *p < 0.05, **p < 0.001 (Obese CAD group vs.controls). Data are mean ± SD.

The recent insight that visceral fat contributes to the development of atherosclerosis constitutes a major breakthrough in understanding the mechanisms underlying these conditions. It is well known that glucocorticoids can influence the development of cardiovascular disease although the process is complex. Glucocorticoids possess a wide range of functions including the stimulation of both glucocorticoid and mineralocorticoid receptors [24], the mediation of opposing actions depending on steroid concentration, and the regulation of systemic cardiovascular risk factors as well as the functional and structural properties of cardiac, vascular and inflammatory cells. This contributes to the difficulty in determining which of these mechanisms involving glucocorticoids contributes to the regulation of cardiovascular disease pathogenesis. 11β-HSD-1 is widely expressed, particularly in liver, adipose tissue, brain, lung, gonads and adrenal cortex [15]. Previous studies suggested that 11β-HSD-1 has an important role in regulating the HPA axis due to its ability of locally amplify glucocorticoids, and its expression in areas known to be relevant to glucocorticoid regulation of HPA axis control. Transgenic mice overexpressing 11β-HSD-1 in adipose tissue develop visceral obesity, insulin resistance, hyperglycemia, dyslipidemia, and hypertension [17]. Global deletion of 11β-HSD-1 caused reduced visceral fat accumulation and these mice had improved insulin sensitivity on a high fat diet [25,26]. In humans, 11β-HSD-1 gene expression in cardiac adipose tissue samples showed the important role of EAT compared with SAT in CAD by inducing local cortisol reactivation [27]. An increase in GCR expression in epicardial fat was reported in obesity and CAD, possibly leading to an amplification of glucocorticoid signaling and growth of this ectopic fat depot [20]. Other studies also demonstrated that visceral adipose tissues expressed high levels of 11β-HSD-1 in obese CAD group, and this is known to be positively associated with metabolic risk factors and CAD [10]. Two studies demonstrated 11β-HSD-1 activity in intact fragments of human omental fat was significantly correlated with visceral fat mass [28,29]. In light of these previous findings, we have investigated the contributory role of the expression of 11β-HSD-1 and GCR in two cardiac visceral fat depots EAT and MAT, in comparison with SAT in the development of atherosclerosis in obese CAD patients. Firstly, we confirm the role of visceral fat depots, especially MAT, in the development of cardiovascular diseases [7]. In addition, we determined a positive correlation between 11β-HSD-1 and GCR gene expression in MAT of obese CAD patients, which would imply an upregulation of glucocorticoid production through 11β-HSD-1 in MAT possibly resulting in an increased volume of MAT in obese CAD patients compared to controls. Although, previous studies reported the altered expressions of the inflammatory adipocytokines (TNF-α, IL-6, leptin and chemerine and adiponectin) in EAT of CAD [30-32], a strong association of MAT and CAD was recently reported by various researchers, and, MAT became the most reliable marker for the cardiometabolic risk [6]. Therefore gene expression results demonstrating increased 11β-HSD-1, GCR, CD68, and inflammation in MAT of obese CAD group might strengthen and contribute to the recently demonstrated MRI data suggesting that MAT volume is a better predictor for CAD compared to other visceral fat depots. Despite the fact that MAT is not close to the coronary arteries, it constitutes the majority of cardiac fat, therefore it is far from being just an inner compartment of intrathoracic fat. Mediastinal adipose tissue (paracardial or intrathoracic) constitutes the majority of cardiac fat accumulation (70%), with EAT constituting approximately 30%. Paracardial fat volume was reported to be nearly twice as high in CAD patients with high-risk coronary lesions as compared to those without CAD, and it was significantly associated with high risk coronary lesion morphology independent of clinical characteristics and general obesity [33,34]. The correlation of intrathoracic and pericardial fat with body mass index and vascular calcification and their association with the presence and severity of coronary artery calcium were also demonstrated [10,35]. In a recent study performed with obese healthy volunteers, increased cardiac fat in MAT (pericardial area) was strongly associated with features of the metabolic syndrome, whereas no correlation was found with EAT [6], and in another study EAT thickness was shown to be useful to predict MS and CAD in patients with BMI < 27 kg/m^2^ but not in obese patients [36]. Alltogether these results indicate the prevalent role of MAT rather than EAT in better diagnosis of cardiometabolic risk in obese patients. Along with the adipokines, increased levels of FFA and the recruitment of macrophages into the adipose tissues were shown to stimulate the inflammation and to impair some transcriptional cascades which eventually lead to the formation of coronary atherosclerosis [37-40]. Therefore, we investigated the correlations of macrophage infiltration and the fatty acids with the gene expressions in visceral fat depots. With the use of CD68 as a macrophage marker, we demonstrated that expression levels of CD68 in EAT and MAT were significantly higher in obese CAD group compared to controls (Figure 1C), and also MAT CD68 was significantly higher compared to EAT and SAT in obese CAD group. In this study, we confirm the presence of macrophage infiltration in EAT of CAD patients which was previously demonstrated by Hirata et al. [11]. Furthermore, as a new finding, we demonstrated the increased gene expression of CD68 in MAT by qRT-PCR and the immunohistochemical staining which would clearly indicate the presence of infiltrating macrophages in this visceral fat in obese CAD group. As the expression is higher compared to EAT and SAT, one might expect a high number of infiltrated macrophages into MAT, and this is quite possible as MAT constitutes the largest volume of the cardiac adipose tissue. Interestingly, in females of obese CAD group, 11β-HSD-1 expression in MAT was strongly correlated with GCR expression and plasma cortisol was found to be positively correlated with 11β-HSD-1 expression. Subtle changes in cortisol were previously reported to be present in obesity. In particular, 11β-HSD-1 was shown to result in higher intraadipose cortisol levels in obese subjects [41], although some studies reported the opposite [42]. Although not statistically significant, cortisol levels were found to be higher in women of obese CAD group compared with men (Data not shown). It was previously described that the cortisol stimulates the mineralocorticoid receptor (MR) in obese patients and in patients with Cushing’s disease via a mechanism involving the saturation of the enzyme 11 hydroxysteroid dehydrogenase. In addition MRs facilitate the expression of inflammatory adipokines and stimulate the proadipogenic effects of glucocorticoid and aldosterone. The inhibition of these receptors increase the expression adiponectin in heart and adipose tissues. To understand the role of increased cortisol on the expression of MR and the associated inflammatory cytokines, we have further studied the mRNA levels of MR along with TNF-α, IL-6 and adiponectin in EAT, MAT and SAT. The results showed that MR mRNA levels were significant increased only in EAT and MAT of obese CAD group compared to controls. There were significant differences in TNF-α, IL-6 and adiponectin mRNA levels between EAT, MAT and SAT of obese CAD group compared to controls (Figure 4). TNF-α and IL-6 were significantly increased in obese CAD group compared to controls, though adiponectin was found to be significantly decreased. These additional findings might suggest that increased cortisol may also interact with MR. In the presence of steroids (aldosterone or glucocorticoids) they would increase the visceral adipogenesis and macrophage infiltration which are known to be the two essential factors in the development of inflammation in the adipose tissue.

Further analysis of the patient group revealed that the expression of 11β-HSD-1 and GCR in EAT, MAT and SAT depots was higher in obese CAD group in both women and men. However the expression of 11β-HSD-1 and GCR in SAT was found to be significantly higher in the male obese CAD group when compared with women. This is also in concordance with Paulsen et al. who reported the effects of gender and sex hormones on 11β-HSD-1 expression in human adipose tissue [43].

### Fatty acids and cardiac adipose tissues: steraridonic acid is positively correlated with mediastinal 11β-HSD-1 expression

Our analysis of fatty acid levels in serum and adipose tissues revealed that stearidonic acid and the n-6/n-3 ratio in EAT and MAT, and palmitoleic acid, eicosatrienoic acid and n-3 in EAT were significantly different in obese CAD group compared to controls. Previous studies have reported that eicosapentaenoic acid supplementation can reduce the risk of coronary heart disease and that it can be synthesized from α-linolenic acid (C18:3n-3) via the conversion of the Δ6-desaturation of α-linolenic acid into stearidonic acid. Interestingly, in EAT and MAT, controls showed significantly lower levels of stearidonic acid compared to obese patients with CAD, moreover arachidonic acid levels were significantly higher in male compared with female obese CAD patients in MAT. Interestingly, the significant expression of genes related with the arachidonic pathway in EAT was recently reported in a whole-genome gene expression microarray analysis of the EAT, MAT and SAT of six male patients suffering from CAD [44]. Our results confirm the expression of arachidonic acid in EAT and show for the first time its significant expression in MAT of obese male CAD patients. In EAT, palmitoleic acid was found to be positively correlated with GCR mRNA expression and in MAT, stearidonic acid strongly correlated with waist circumference and 11β-HSD-1 MAT. Recently, Vara Prasad et al. showed that, in rats, trans fatty acids and saturated fatty acids increase local amplification of glucocorticoid activity in adipose tissue and that this increase occurs by upregulation of 11β-HSD1 following changes in the expression of its main transcription factor C/EBP-α [45]. In obese CAD patients, we confirm increased presence of stearidonic acid and arachidonic acid in cardiac visceral fat.

Together, our findings demonstrate that MAT is closely associated with 11β-HSD-1 mRNA expression in obese CAD group. Interestingly, our findings also suggest that inflammatory cell infiltration is enhanced in the MAT of obese patients with CAD, highlighting the influential role of chronic inflammation in MAT on the pathogenesis of coronary atherosclerosis.

To our knowledge, this is the first study describing a positive correlation between 11β-HSD-1 and GCR in MAT, and it seems likely that 11β-HSD-1 and GCR directly regulate each other. The regression analysis results revealed that GCR mRNA levels in MAT, cortisol, stearidonic acid and HOMA-IR explained 40.2% of the variance in 11β-HSD-1 mRNA levels in MAT, while MAT 11β-HSD-1 mRNA levels and cortisol explained 35.1% of the variance in MAT GCR mRNA levels, MAT 11β-HSD-1 and GCR mRNA levels and cortisol explained 56.4% variance in MAT CD68 mRNA levels. Along with the increased cortisol levels, expression of MR and the inflammatory cytokines were also found to be increased whereas the expression of adiponectin was decreased in MAT of obese CAD group. These results may also provide insight into the biology of MAT and potentially suggest a degree of cross-talk between glucocorticoid action, cortisol, fatty acids, macrophages, inflammation and mediastinal adipose tissue in obese CAD group. The differential expression of fatty acids in adipose tissues, especially stearidonic acid in EAT and MAT, may reflect differences in the functional mechanisms of fatty acid transporters in each adipose tissue, and therefore our preliminary results might well be used in future studies to determine these mechanisms.

## Conclusion

In conclusion, we report for the first time increased expression of 11β-HSD-1, GCR and CD68 in MAT and also demonstrated the interrelated effects of biological parameters, fatty acids, along with related gene expressions to explain the variance of 11β-HSD-1, GCR and CD68 expressions in MAT of obese CAD patients. These findings support the hypothesis that MAT contributes locally to the development of coronary atherosclerosis via the glucocorticoid action.

## Abbreviations

MAT: Mediastinal adipose tissue; EAT: Epicardial adipose tissue; SAT: Subcutaneous adipose tissue; CAD: Coronary artery disease; 11β-HSD-1: 11beta-hydroxysteroid dehydrogenase type 1; GCR: Glucocorticoid receptor; CABG: Coronary artery bypass grafting; FFA: Free fatty acid.

## Competing interests

The authors declare no conflict of interest.

## Authors’ contributions

ASB and FA designed the study. BC and BA did the cardiac surgeries. SG and ASB consulted the patients. FA and GA performed the molecular analysis. CD performed and analyzed the CT scans of the study group. DG performed the biochemical analysis. ZY performed the fatty acids analysis. GT and SBG performed the immunohistochemical staining and analysis. FA, SG, UO and ZY analyzed the data. FA drafted the manuscript. All authors contributed to the final version of the manuscript.

## Funding

This work was supported by Turkish Diabetes Foundation.
